# Supplementary material for: Long-Term, Sex-Specific Effects of GCRsim and Gamma Irradiation on the Brains, Hearts, and Kidneys of Mice with Alzheimer’s Disease Mutations
Source: Int J Mol Sci. 2024 Aug 16;25(16):8948. doi: 10.3390/ijms25168948 (PMC11355020; doi:10.3390/ijms25168948)
Supplement: Supplementary file 1 [file ijms-25-08948-s001.zip › ijms-3121576-supplementary.pdf]

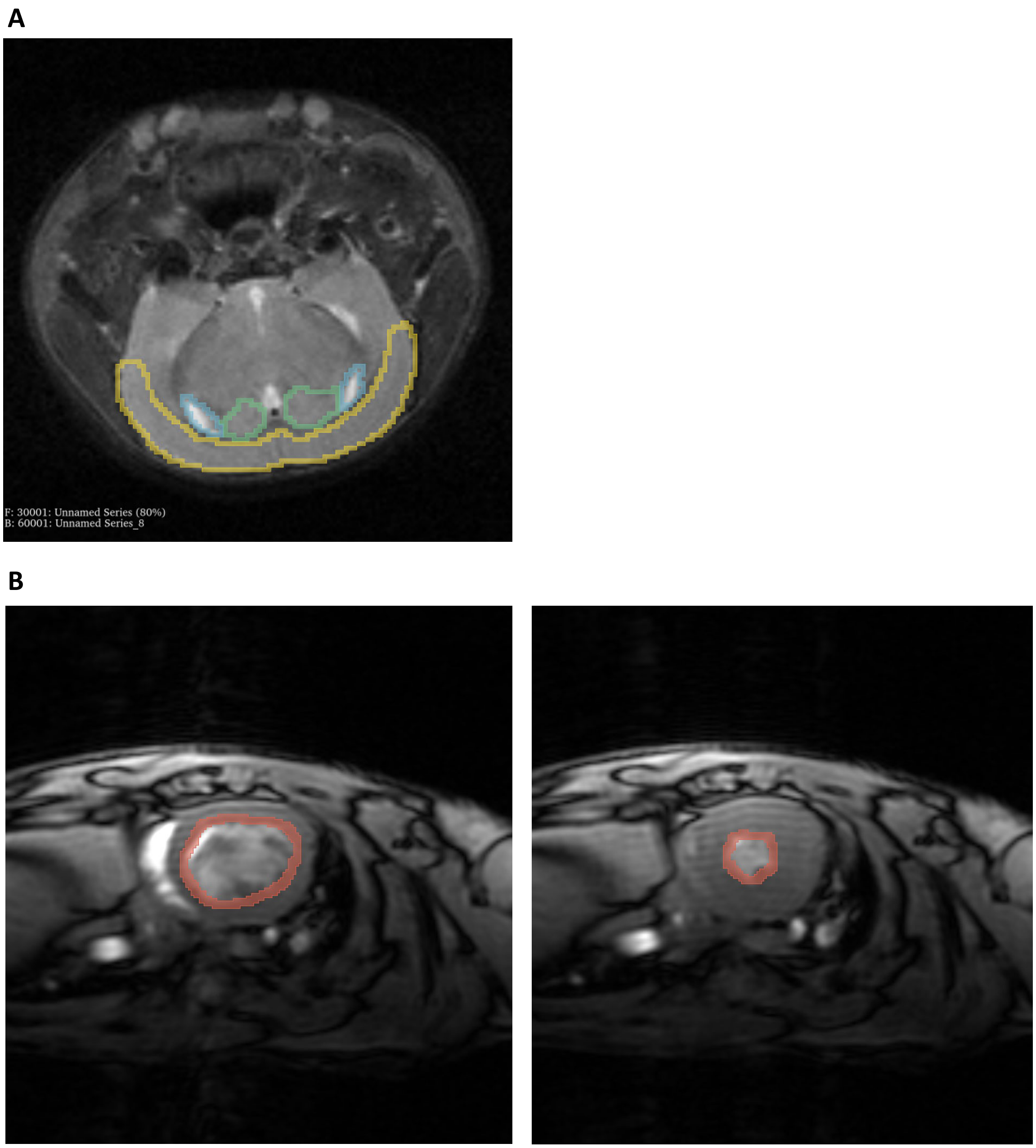

Supplemental Figure S1. (**A**): Brain MRI image from a 0.75 Gy GCRsim irradiated M Tg mouse. Yellow segmentation outlines the cortex, blue segmentation outlines the lateral ventricles, and purple segmentation outlines the hippocampus. (**B**): MRI images of the heart from the same mouse. The image on the left represents end diastole, whereas the image on the right shows end systole. Red segmentation outlines the volume of the ventricle at these two phases.

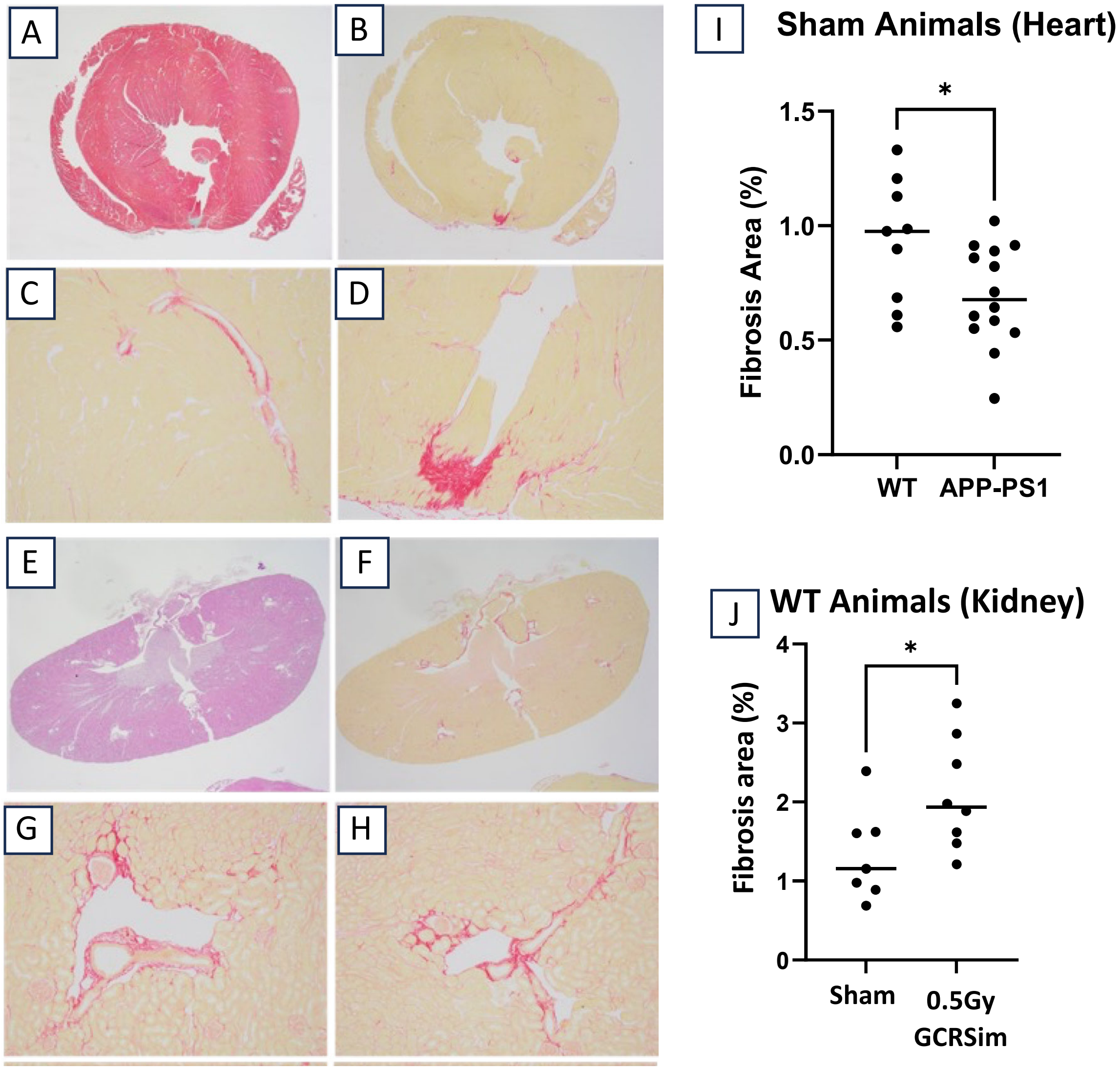

Supplemental Figure S2. (**A**-**D**) Images of heart sample from a 0.75 Gy gamma F WT mouse. Fibrosis = 3.433%. Masson's trichrome, (**A**); PSR (**B** – **D**). 2x (**A**, **B**); 10x (**C** – **D**). (**E**-**H**) Images of kidney sample from a 2 Gy gamma M WT mouse. Fibrosis = 3.133%. H&E, (**E**); PSR (**F** – **H**). 1.25x (**E**, **F**); 10x (**G**, **H**). (**I**) Comparison between WT vs APP/PS1 sham animals. (**J**). Comparison between WT animals, sham and irradiated (low-dose GCRSim).

Supplementary Table S1: Summary of statistically significant differentially expressed genes in mouse heart and kidney

Tissue	Target	Comparison	p.adjust	FoldChange	FCMax	FCMin
Heart	Casp3	APP-PS1(sham) vs WT(sham)	0.0016	2.904307659	40.55357726	0.207996521
Heart	GLUT4	APP-PS1(sham) vs WT(sham)	0.044	1.664986965	11.36065299	0.244016044
Heart	Trp53	APP-PS1(sham) vs WT(sham)	0.046	2.511406628	53.49955809	0.117891876
Heart	Col1A1(c)	WT(0.5 Gy GCRsim) vs WT(sham)	0.018	2.097541488	8.864319657	0.496335925
Heart	MCAD	WT(0.5 Gy GCRsim) vs WT(sham)	0.022	0.358088092	5.065733092	0.025312641
Heart	TGF-B1(b)	WT(0.5 Gy GCRsim) vs WT(sham)	0.041	0.711029181	2.604125467	0.19413907
Heart	VLCAD	WT(0.5 Gy GCRsim) vs WT(sham)	0.00052	0.493234733	0.880627341	0.276258175
Heart	Casp3	WT(0.75 Gy GCRsim) vs WT(sham)	0.00026	0.602281659	1.556184489	0.23309781
Heart	Col1A1(c)	WT(0.75 Gy GCRsim) vs WT(sham)	0.0031	2.630592838	13.53024363	0.511448195
Heart	CTGF	WT(0.75 Gy GCRsim) vs WT(sham)	0.043	0.766217987	2.004680976	0.292859567
Heart	GLUT4	WT(0.75 Gy GCRsim) vs WT(sham)	1.70E-05	0.577388739	1.159855967	0.287430307
Heart	TGF-B1(b)	WT(0.75 Gy GCRsim) vs WT(sham)	0.0034	0.665560523	1.883705665	0.235159249
Heart	VCAM-1	WT(0.75 Gy GCRsim) vs WT(sham)	0.018	0.780357853	1.59041247	0.382893362
Heart	VLCAD	WT(0.75 Gy GCRsim) vs WT(sham)	0.00037	0.441768091	0.979736367	0.19919547
Heart	Casp3	WT(0.75 Gy GCRsim) vs WT(0.5 Gy GCRsim)	0.01	0.632255515	1.633631395	0.244698429
Heart	MCAD	WT(0.75 Gy GCRsim) vs WT(0.5 Gy GCRsim)	3.20E-05	5.075792674	20.58627293	1.251497605
Heart	Trp53	WT(0.75 Gy GCRsim) vs WT(0.5 Gy GCRsim)	0.0018	0.600907511	1.436142651	0.251430341
Heart	Col1A1(c)	WT(0.75 Gy gamma) vs WT(sham)	0.0038	2.706368421	12.62789619	0.580019816
Heart	ICAM-1	WT(0.75 Gy gamma) vs WT(sham)	0.034	1.69177567	5.007407455	0.571574201
Heart	OSTP	WT(0.75 Gy gamma) vs WT(sham)	0.025	1.727421572	6.730538757	0.443350138
Heart	Trp53	WT(0.75 Gy gamma) vs WT(sham)	0.039	1.678595566	3.884163451	0.725428554
Heart	BMP4	WT(2.0 Gy gamma) vs WT(sham)	0.049	1.696105384	10.00522429	0.287527135
Heart	ICAM-1	WT(2.0 Gy gamma) vs WT(sham)	0.032	1.62977215	3.034951435	0.875189379
Heart	Col1A1(c)	WT(2.0 Gy gamma) vs WT(0.75 Gy gamma)	7.70E-06	0.248253811	0.944200943	0.065272075
Heart	BMP4	APP-PS1(0.5 Gy GCRsim) vs APP-PS1(sham)	0.014	0.636825744	1.955699623	0.207366726
Heart	Casp3	APP-PS1(0.5 Gy GCRsim) vs APP-PS1(sham)	0.009	0.37892715	1.576057164	0.091104427
Heart	CTGF	APP-PS1(0.5 Gy GCRsim) vs APP-PS1(sham)	0.0021	0.661399223	1.907329143	0.229351569
Heart	OSTP	APP-PS1(0.5 Gy GCRsim) vs APP-PS1(sham)	1.50E-05	0.398761028	1.147704126	0.138546472
Heart	VCAM-1	APP-PS1(0.5 Gy GCRsim) vs APP-PS1(sham)	0.035	0.782069995	1.444011931	0.423565391
Heart	VLCAD	APP-PS1(0.5 Gy GCRsim) vs APP-PS1(sham)	0.0025	0.654613441	0.899361547	0.476469957
Heart	Casp3	APP-PS1(0.75 Gy GCRsim) vs APP-PS1(sham)	0.00024	0.273455692	0.662365352	0.112895422
Heart	CTGF	APP-PS1(0.75 Gy GCRsim) vs APP-PS1(sham)	0.00033	0.577229594	1.965323877	0.169536435
Heart	GLUT4	APP-PS1(0.75 Gy GCRsim) vs APP-PS1(sham)	0.00083	0.415310954	0.903760126	0.190850629
Heart	OSTP	APP-PS1(0.75 Gy GCRsim) vs APP-PS1(sham)	0.041	0.626071218	2.097409303	0.186880629
Heart	TGF-B1(b)	APP-PS1(0.75 Gy GCRsim) vs APP-PS1(sham)	0.00015	0.368208666	1.879980834	0.072116492
Heart	VLCAD	APP-PS1(0.75 Gy GCRsim) vs APP-PS1(sham)	0.0014	0.570686153	0.878855674	0.370575847
Heart	BMP4	APP-PS1(0.75 Gy GCRsim) vs APP-PS1(0.5 Gy GCRsim)	0.012	1.668944438	5.411554659	0.514708936
Heart	GLUT4	APP-PS1(0.75 Gy GCRsim) vs APP-PS1(0.5 Gy GCRsim)	0.045	0.697545326	1.517931674	0.320547684
Heart	MCAD	APP-PS1(0.75 Gy GCRsim) vs APP-PS1(0.5 Gy GCRsim)	0.0011	4.053627917	23.19524343	0.708416764
Heart	OSTP	APP-PS1(0.75 Gy GCRsim) vs APP-PS1(0.5 Gy GCRsim)	0.029	1.570041136	5.259815165	0.468653193
Heart	TGF-B1(b)	APP-PS1(0.75 Gy GCRsim) vs APP-PS1(0.5 Gy GCRsim)	0.0015	0.425799813	2.174026742	0.083396159
Heart	VCAM-1	APP-PS1(0.75 Gy GCRsim) vs APP-PS1(0.5 Gy GCRsim)	0.0015	1.499369601	3.264062388	0.688745782
Kidney	BMP4	APP-PS1(sham) vs WT(sham)	0.0015	0.637083494	1.372669683	0.29568321
Kidney	BMP4	WT(0.75 Gy GCRsim) vs WT(sham)	0.039	0.74653937	1.670589641	0.333607379
Kidney	CTGF	WT(0.75 Gy GCRsim) vs WT(sham)	0.00096	0.65669086	1.128957917	0.381983135
Kidney	MCAD	WT(0.75 Gy GCRsim) vs WT(sham)	0.019	1.595126882	3.825219941	0.66517215
Kidney	Trp53	WT(0.75 Gy GCRsim) vs WT(sham)	0.024	0.802771388	1.395942424	0.46165364
Kidney	CTGF	WT(0.75 Gy GCRsim) vs WT(0.5 Gy GCRsim)	0.00037	0.734763581	1.263177566	0.42739638
Kidney	ICAM-1	WT(0.75 Gy GCRsim) vs WT(0.5 Gy GCRsim)	0.029	1.224784279	2.235839363	0.670932159
Kidney	Trp53	WT(0.75 Gy gamma) vs WT(sham)	0.0097	0.769499528	1.398262323	0.423475276
Kidney	Casp3	WT(2.0 Gy gamma) vs WT(sham)	0.032	0.799268	1.427400325	0.447547422
Kidney	GLUT4	WT(2.0 Gy gamma) vs WT(sham)	0.016	0.791563916	1.37149057	0.456855808
Kidney	ICAM-1	WT(2.0 Gy gamma) vs WT(0.75 Gy gamma)	0.00078	1.376853455	2.575471171	0.736069368
Kidney	BMP4	APP-PS1(0.5 Gy GCRsim) vs APP-PS1(sham)	0.0072	1.450572661	3.154559511	0.667022143
Kidney	Casp3	APP-PS1(0.5 Gy GCRsim) vs APP-PS1(sham)	0.049	0.811767495	1.254644326	0.525221733
Kidney	CTGF	APP-PS1(0.5 Gy GCRsim) vs APP-PS1(sham)	0.0053	0.785919071	1.19720314	0.515926467
Kidney	MCAD	APP-PS1(0.5 Gy GCRsim) vs APP-PS1(sham)	0.038	1.511098932	3.608746098	0.63274609
Kidney	TGF-B1(b)	APP-PS1(0.5 Gy GCRsim) vs APP-PS1(sham)	0.041	1.358077434	2.916201638	0.632457746
Kidney	Trp53	APP-PS1(0.5 Gy GCRsim) vs APP-PS1(sham)	0.00044	0.710026094	1.120492858	0.449924379
Kidney	BMP4	APP-PS1(0.75 Gy GCRsim) vs APP-PS1(sham)	0.0012	1.563639121	3.336515267	0.732790683
Kidney	Casp3	APP-PS1(0.75 Gy GCRsim) vs APP-PS1(sham)	0.049	0.757730366	1.77226021	0.323967838
Kidney	Col1A1(c)	APP-PS1(0.75 Gy GCRsim) vs APP-PS1(sham)	0.007	0.589747965	1.394346965	0.249437673
Kidney	CTGF	APP-PS1(0.75 Gy GCRsim) vs APP-PS1(sham)	0.0048	0.77251162	1.253136131	0.47622456
Kidney	ICAM-1	APP-PS1(0.75 Gy GCRsim) vs APP-PS1(sham)	0.00079	1.590176109	3.588410908	0.704674053
Kidney	MCAD	APP-PS1(0.75 Gy GCRsim) vs APP-PS1(sham)	0.015	1.587304965	3.425974015	0.735422114
Kidney	TGF-B1(b)	APP-PS1(0.75 Gy GCRsim) vs APP-PS1(sham)	0.024	1.419952668	3.359198065	0.600222297
Kidney	Col1A1(c)	APP-PS1(0.75 Gy GCRsim) vs APP-PS1(0.5 Gy GCRsim)	0.0013	0.460808002	1.089492931	0.194901691
Kidney	ICAM-1	APP-PS1(0.75 Gy GCRsim) vs APP-PS1(0.5 Gy GCRsim)	0.00077	1.51226234	3.41258974	0.670147178
